# Antimicrobial Resistance and Genomic Characteristics of *Escherichia coli* Strains Isolated from the Poultry Industry in Henan Province, China

**DOI:** 10.3390/microorganisms12030575

**Published:** 2024-03-13

**Authors:** Zugang Li, Chaoying Jia, Zizhe Hu, Yancheng Jin, Tianzhi Li, Xiaoxue Zhang, Zhong Peng, Ruicheng Yang, Huanchun Chen, Xiangru Wang

**Affiliations:** 1 National Key Laboratory of Agricultural Microbiology, College of Veterinary Medicine, Huazhong Agricultural University, Wuhan 430070, China; lizugang@webmail.hzau.edu.cn (Z.L.); jcy116178@webmail.hzau.edu.cn (C.J.); huzizhede@webmail.hzau.edu.cn (Z.H.); jinyancheng@webmail.hzau.edu.cn (Y.J.); litianzhi@webmail.hzau.edu.cn (T.L.); zhangxuenian@webmail.hzau.edu.cn (X.Z.); pengzhong@webmail.hzau.edu.cn (Z.P.); yangruicheng@webmail.hzau.edu.cn (R.Y.); chenhch@webmail.hzau.edu.cn (H.C.); 2 Key Laboratory of Preventive Veterinary Medicine in Hubei Province, The Cooperative Innovation Center for Sustainable Pig Production, Wuhan 430070, China; 3 Key Laboratory of Development of Veterinary Diagnostic Products, Ministry of Agriculture of the People’s Republic of China, Wuhan 430070, China; 4 International Research Center for Animal Disease, Ministry of Science and Technology of the People’s Republic of China, Wuhan 430070, China

**Keywords:** antimicrobial susceptibility testing, multidrug resistance, sequence types, antimicrobial resistance genes

## Abstract

*Escherichia coli* (*E. coli*) is an important foodborne pathogen and a biomarker for monitoring antimicrobial resistance. Investigating the prevalence of *E. coli* in the poultry industry holds great importance, particularly in Henan province, a major poultry-producing region in China. Here, we investigated the antimicrobial resistance (AMR) phenotypes of *E. coli* strains obtained from the poultry industry in Henan, China. A total of 344 *E. coli* strains were isolated from 638 samples collected from seven farms, three slaughterhouses, and ten terminal markets. Approximately 96.4%, 81.7%, and 52.5% of the isolates from the farms, slaughterhouses, and terminal markets exhibited multidrug resistance. Whole-genome sequencing was performed on 169 strains to reveal their genomic characteristics. The sequence type (ST) analysis revealed that ST10 and ST156 were the most frequent types within the poultry supply chain, whereas ST10 and ST162 were commonly found across the farms, slaughterhouses, and terminal markets. Fourteen ST10 *E. coli* strains belonged to phylogenetic group A, while fifteen ST165 and six ST162 *E. coli* strains belonged to phylogenetic group B1. In addition, several antimicrobial resistance genes and virulence factor genes were identified. The *bla*_NDM-5_ gene mediated carbapenem resistance in two *E. coli* strains, while *mcr-1*-mediated colistin resistance was detected in nine *E. coli* strains. Phylogenetic group A exhibited fewer virulence genes compared to other groups of *E. coli*. Plasmid replicons, such as IncFIB (AP001918), IncX1, IncFIC (FII), and IncFII (pHN7A8), were frequently observed. These findings provide valuable insights into the current AMR profiles of *E. coli* strains isolated from the poultry industry in Central China and highlight the need to implement good manufacturing practices and reduce antibiotic usage to mitigate potential risks associated with *E. coli*.

## 1. Introduction

Antimicrobials were previously employed in livestock for growth promotion and disease control [1]. However, their widespread use in animals has accelerated the emergence of antibiotic-resistant bacteria, which can cause severe public health problems [2]. *Escherichia coli* (*E. coli*) is an important foodborne pathogen, and foodborne diseases caused by this agent result in high mortality and morbidity rates in different regions of the world [3,4,5]. Additionally, *E. coli* has been recognized as a natural reservoir of antimicrobial resistance genes (ARGs), and it has an important role in the spread of antimicrobial resistance (AMR) [2,6]. Therefore, it is widely used as an essential biomarker for monitoring AMR [7,8]. In recent years, whole genome sequencing (WGS) has played an important role in the rapid discovery of the resistance mechanism and epidemic characteristics of *E. coli*. For example, it has been utilized in England to investigate food-borne outbreaks of *E. coli* O157:H7 [9] and in China for the surveillance of *E. coli* AMR in pig farms [10].

Poultry is an important source of proteins for humans, and the consumption of poultry is increasing annually in China (http://www.stats.gov.cn/, accessed on 1 January 2023). Henan province is an important poultry-producing province in China, with nearly 500 million domestic birds slaughtered as of 2021 (http://www.hnsmeat.cn/index.html, accessed on 16 February 2022). Avian pathogenic *E. coli* is reportedly the most common bacterial pathogen that infects chickens, costing the poultry industry hundreds of millions of dollars in economic losses [11]. Accordingly, monitoring foodborne pathogen contaminations in different nodes of the poultry supply chain in this region has great significance [12,13,14]. To our knowledge, there seems to be a lack of data on the prevalence of *E. coli* in the poultry industry in Henan province, China. In this study, we isolated *E. coli* from broiler farms, slaughterhouses, and terminal markets in three separate poultry supply chains in Henan province, China. To further understand AMR and its prevalence in *E. coli*, the aim of this study was to provide a profile of AMR phenotypes and genotypes.

## 2. Materials and Methods

### 2.1. Sample Collection and Bacterial Isolation

**Figure 1:**
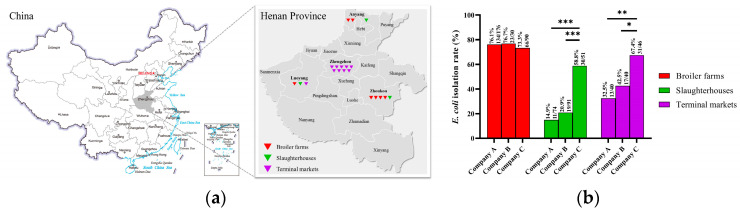
Sampling sites and isolation of bacteria: (**a**) map showing the sampling sites for the broiler farms, slaughterhouses, and markets in Henan province. These maps are not drawn to scale. (**b**) *Escherichia coli* isolation rate (number of *E. coli*/samples) from companies A, B, and C. The results were analyzed using the chi-square test, and (*) means significant at a *p*-value < 0.05, (**) means significant at a *p*-value < 0.01, and (***) means significant at a *p*-value < 0.001.

Between April and August 2019, 638 samples were collected from seven broiler farms, three slaughterhouses, eight retail markets, one supermarket, and one wholesale market in four cities in Henan province (Figure 1a). In total, 296 samples were collected from broiler farms (from cotton swabs of the cloaca or cecum contents of dead broilers, the cloaca of healthy broilers, and the feces, floor, cage, feed, and drinking water), 216 samples were collected from slaughterhouses (from trays, conveyor belts, knives, chopping boards, the floor, the shelves, and organs), and 126 samples were collected from markets (meat and organs). Samples, after enrichment culture, were streaked directly onto MacConkey Agar (Hopebiol, Qingdao, China) for the selection of *E. coli* strains, following the protocol established by Hu et al., which aligns with our National Standards for *E. coli* strain isolation and identification [15]. The single colony was streaked again onto Eosin-Methylene Blue Agar (Hopebiol, Qingdao, China) for the further purification of the *E. coli* strains. Morphologically compatible colonies were inoculated in Luria-Bertani (LB; 1% Trytone (Thermo Fisher, Shanghai, China), 0.5% Yeast Extract [Thermo Fisher, Shanghai, China], and 1% NaCl [Sinopharm, Beijing, China]) for enrichment culture. The isolated strains were confirmed through amplification of the partial 16S rDNA gene and seven housekeeping genes (*adk*, *fumC*, *gyrB*, *icd*, *mdh*, *purA*, and *recA*), according to a previously described protocol [16,17]. The primers used are listed in Table S1.

### 2.2. Antimicrobial Susceptibility Testing

The minimal inhibitory concentrations (MICs) of antibiotics of different classes, against *E. coli* strains, were determined using the broth microdilution method, following the protocols recommended by the Clinical and Laboratory Standards Institute (CLSI) [18]. Nine antimicrobial classes (MedChemExpress, Shanghai, China), including those belonging to the penicillins (ampicillin), cephems (ceftazidime), penems (meropenem, imipenem), aminoglycosides (gentamicin), tetracyclines (tetracycline, tigecycline), lipopeptides (colistin), phenicols (florfenicol), quinolones (ofloxacin), and folate pathway antagonists (trimethoprim–sulfamethoxazole) were included in the tests. *Escherichia coli* ATCC^®^ 25922 was used for quality control. The results were interpreted using CLSI breakpoints [19,20]. If a CLSI breakpoint was not available, the European Committee on Antimicrobial Susceptibility Testing (EUCAST) breakpoints were considered [21,22]. The breakpoints and quality controls for the antimicrobial agents are listed in Table S2.

### 2.3. DNA Extraction, WGS, and Bioinformatic Analysis

Among these *E. coli* strains, 169 isolates, consisting of 122 isolates from broiler farms, 23 isolates from slaughterhouses, and 24 isolates from markets, were selected for WGS. Genomic DNA was extracted using a bacterial genomic DNA Kit (Cwbiotech, Beijing, China), according to the manufacturer’s instructions. The quality and concentration of the bacterial genomic DNA were evaluated by electrophoresis on a 1% agarose gel, using a NanoDrop2000 system (Thermo Scientific, Waltham, MA, USA) and a Qubit 2.0 fluorometer (Thermo Scientific, Waltham, USA). Libraries were constructed based on the qualified DNA using the NEBNext UltraTM II DNA Library Prep Kit (New England BioLabs, Ipswich, MA, USA) and sequenced on an Illumina NovaSeq 6000 platform (Novogene, Tianjin, China). High-quality clean reads were selected for the de novo assembled using SPAdesv3.9.0 to generate genome contigs. The complete nucleotide sequences of these *E. coli* strains were deposited in the NCBI database under the accession number PRJNA767170. The sequence types (STs), O-serogroups, phylogenetic groups, ARGs, virulence factor genes (VFGs), and plasmid types of these confirmed *E. coli* strains were predicted using tandem contigs of the samples based on MLST 2.0 [23], SerotypeFinder 2.0 [24], ClermonTyping [25], ResFinder 4.1 [26], VirulenceFinder 2.0 [27,28], and PlasmidFinder 2.1 [29]. A phylogenetic tree was constructed based on the MLST data using MEGAX [30], using the neighbor-joining algorithm with 1000 bootstraps.

### 2.4. Statistical Analysis

The chi-square test was used to test for significant differences in the prevalence of *E. coli* among the samples collected from the broiler supply chain. A *p*-value < 0.05 (*) was considered significant, and *p* < 0.01 (**) or *p* < 0.001 (***) was considered extremely significant. Statistical analyses were performed using GraphPad Prism 8 (version 8.4.3).

## 3. Results

### 3.1. Prevalence of E. coli

In total, 344 *E. coli* strains were isolated from 638 samples. Of these, 158 *E. coli* strains were isolated from Company A, including 134 strains from four broiler farms, 11 strains from one slaughterhouse, and 13 strains from eight retail markets; 59 *E. coli* strains were isolated from Company B, including 23 strains from one broiler farm, 19 strains from slaughterhouses, and 17 strains from one supermarket; and 127 *E. coli* strains were isolated from Company C, including 66 strains from two broiler farms, 30 strains from one slaughterhouse, and 31 strains from one wholesale market.

There was a significant difference in the *E. coli* isolation rates among the three nodes, and *E. coli* was more prevalent in the samples collected from broiler farms (75.3%) than in those collected from slaughterhouses (27.8%, *p* < 0.001) and terminal markets (48.4%, *p* < 0.001). Similar results were found for all three companies. There was no significant difference in the rates of *E. coli* isolation from the broiler farms. The rates of *E. coli* isolation in both the slaughterhouse and terminal markets of Company C were significantly higher than those of Companies A and B (Figure 1b).

### 3.2. Antimicrobial Resistance Phenotypes of E. coli

**Figure 2:**
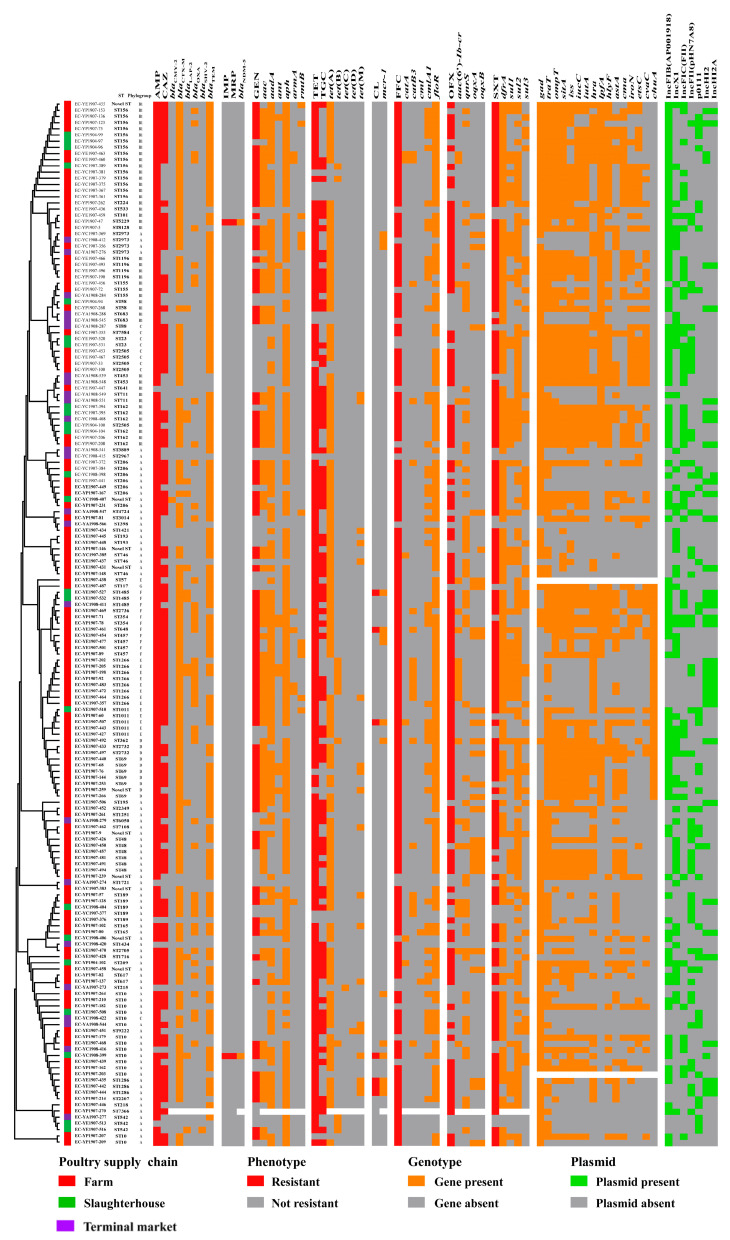
Distribution of the antimicrobial resistance phenotypes, antimicrobial resistance genes, virulence factor genes, and plasmid replicons. The phylogenetic tree was constructed based on multilocus sequence typing (MLST) alleles using MEGAX, as well as the neighbor-joining algorithm with 1000 bootstraps. AMP, ampicillin; CAZ, ceftazidime; IMP, imipenem; MRP, meropenem; GEN, gentamicin; OFX, ofloxacin; SXT, trimethoprim–sulfamethoxazole; TET, tetracycline; TGC, tigecycline; CL, colistin; FFC, florfenicol.

**Figure 3:**
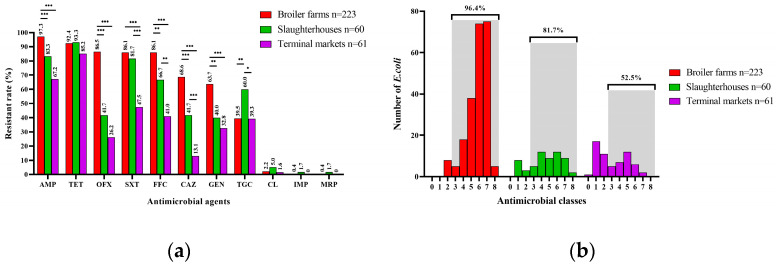
Antimicrobial resistance characteristics of *Escherichia coli* strains isolated from the poultry industry: (**a**) percentages of *E. coli* strains showing antimicrobial resistance to different antimicrobial agents. AMP, ampicillin; CAZ, ceftazidime; IPM, imipenem; MRP, meropenem; GEN, gentamicin; OFX, ofloxacin; SXT, trimethoprim–sulfamethoxazole; TET, tetracycline; TGC, tigecycline; CL, colistin; FFC, florfenicol. (**b**) Percentages of *E. coli* strains showing antimicrobial resistance to different antimicrobial classes. The grey area represents the multidrug-resistant *E. coli* strains. The results were analyzed using a chi-square test, (*) means significant at a *p*-value < 0.05, (**) means significant at a *p*-value < 0.01, and (***) means significant at a *p*-value < 0.001.

The AST results show that isolates from broiler farms and slaughterhouses had severe resistance profiles (Table S3). Many of these *E. coli* strains were resistant to tetracycline (broiler farms: 92.4%, 206/223; slaughterhouses: 93.3%, 56/60; terminal markets: 85.2%, 52/61), ampicillin (broiler farms: 97.3%, 217/223; slaughterhouses: 83.3%, 50/60; terminal markets: 67.2%, 41/61), trimethoprim–sulfamethoxazole (broiler farms: 86.1%, 192/223; slaughterhouses: 81.7%, 49/60; terminal markets: 47.5%, 29/61), florfenicol (broiler farms: 86.1%, 192/223; slaughterhouses: 66.7%, 40/60; terminal markets: 41.0%, 25/61), whereas relatively few *E. coli* strains were resistant to colistin (broiler farms: 2.2%, 5/223; slaughterhouses: 5.0%, 3/60; terminal markets: 1.6%, 1/61), meropenem (broiler farms: 0.4%, 1/223; slaughterhouses: 1.7%, 1/60; terminal markets: 0%), and imipenem (broiler farms: 0.4%, 1/223; slaughterhouses: 1.7%, 1/60; terminal markets: 0%) (Figure 2 and Figure 3a). Approximately 86.0% (296/344) of the *E. coli* strains isolated from broiler farms (96.4%, 215/223), slaughterhouses (81.7%, 49/60), and terminal markets (52.5%, 32/61) were multidrug-resistant (MDR) strains (Figure 3b). Moreover, broiler farms had significantly higher rates of resistance to several antimicrobial agents than slaughterhouses and terminal markets, except slaughterhouses had significantly higher rates of resistance to tigecycline (Figure 3b).

Of note, many *E. coli* strains showed resistance to tigecycline (43.0%, 148/344 of all *E. coli* strains), but a large proportion of them showed MICs ranging from 1 to 2 µg/mL (95.9%, 142/148), a very small proportion of them showed a high level of resistance (MIC ≥ 4 µg/mL; 4.1%, 6/148). In particular, 0.4% (1/191) and 2.2% (5/191) of the broiler farm isolates, and 1.7% (1/60) and 5.0% (3/60) of the slaughterhouse isolates were resistant to carbapenems and colistin, respectively. In addition, 1.6% (1/61) of terminal market isolates were resistant to colistin. Surprisingly, one *E. coli* strain isolated from a slaughterhouse was resistant to carbapenems and colistin.

### 3.3. Sequence Type, Phylogenetic Group and O-Serogroup

**Figure 4:**
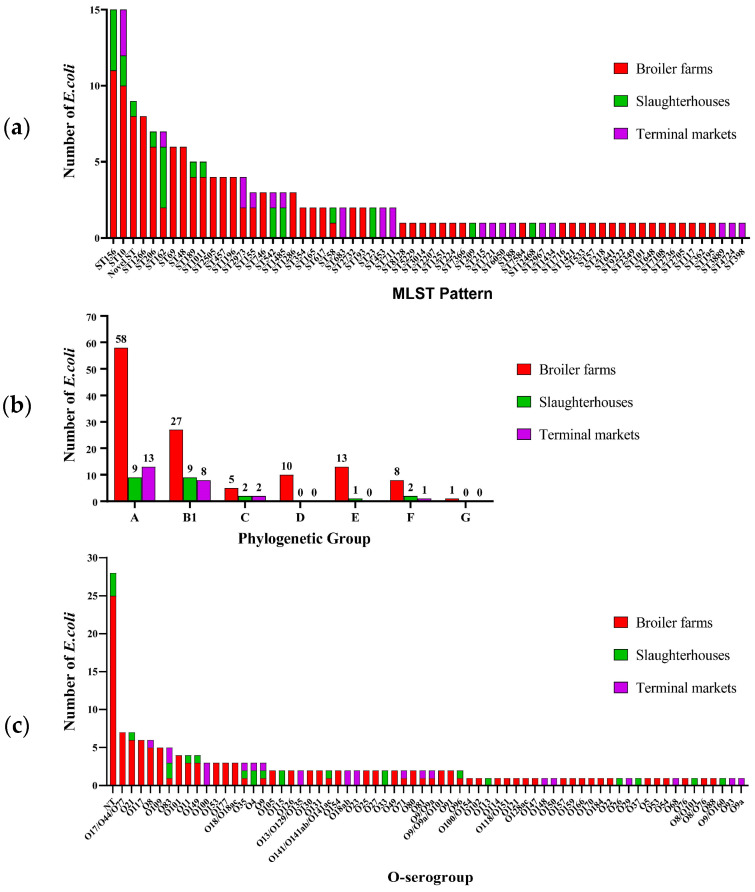
Sequence types, phylogenetic groups, and O-serogroups of 169 *Escherichia coli* isolates from the poultry industry. Distributions of the (**a**) sequence types, (**b**) phylogenetic groups, and (**c**) O-serogroups among 169 *E. coli* strains are shown. MLST, multilocus sequence typing.

In total, 63 catalogs of STs were identified among the 160 *E. coli* strains, and based on the results, a novel ST might exist for 9 *E. coli* strains (Figure 4a). ST156 (8.9%, 15/169) and ST10 (8.9%, 15/169) were the most common STs in the poultry industry, followed by ST1266 (4.7%, 8/169). Further, ST10 and ST162 were the most commonly identified STs among the isolates from broiler farms, slaughterhouses, and terminal markets. In total, 46 catalogs of STs were identified in broiler farm isolates, and ST156 (9.0%, 11/122) was the most frequent. Moreover, 12 total catalogs of STs were identified in slaughterhouse isolates, with ST156 (17.4%, 4/23) and ST162 (17.4%, 4/23) being the most frequent. In total, 18 catalogs of STs were identified in terminal market isolates, with ST10 (12.5%, 3/24) being the most frequent. ST156, ST206, ST189, ST1011, and ST58 were the most commonly identified STs among the isolates from broiler farms and slaughterhouses, whereas ST2973, ST155, ST542, and ST1485 were also identified among the isolates from slaughterhouses and terminal markets (Figure 4a).

The phylogenetic analysis showed that 47.3% (80/169), 26.0% (44/169), 5.3% (9/169), 5.9% (10/169), 8.3% (14/169), 6.5% (11/169), and 0.6% (1/169) of poultry industry isolates belonged to phylogenetic groups A, B1, C, D, E, F, and G, respectively (Figure 4b). Moreover, isolates from phylogenetic groups A, B1, C, and F were commonly identified among the isolates from broiler farms, slaughterhouses, and terminal markets. A relatively large proportion of ST10 *E. coli* strains belonged to group A (93.3%, 14/15), whereas all 15 ST165 and six ST162 *E. coli* strains belonged to phylogenetic group B1. In addition, they were all MDR *E. coli* strains.

The diversity of O-serogroups was confirmed in the poultry industry isolates, and 69.8% (118/169) of these *E. coli* strains were O-antigen typable and classified into 56 O-serogroup catalogs; however, 16.6% (28/169) of these *E. coli* strains were O-antigen non-typable (Figure 4c, Table S4). Multiple serogroups were predicted in 13.6% (23/169) of the *E. coli* strains. The dominant O-serogroups among the isolates from the poultry industry were O21 (4.1%, 7/169), O117 (3.6%, 6/169), and O8 (3.6%, 6/169). In addition, the O-serogroups O83, O35, and O9 were commonly identified among isolates from broiler farms, slaughterhouses, and terminal markets.

### 3.4. Prevalence of Antimicrobial Resistance Genes and Virulence Factor Genes

**Figure 5:**
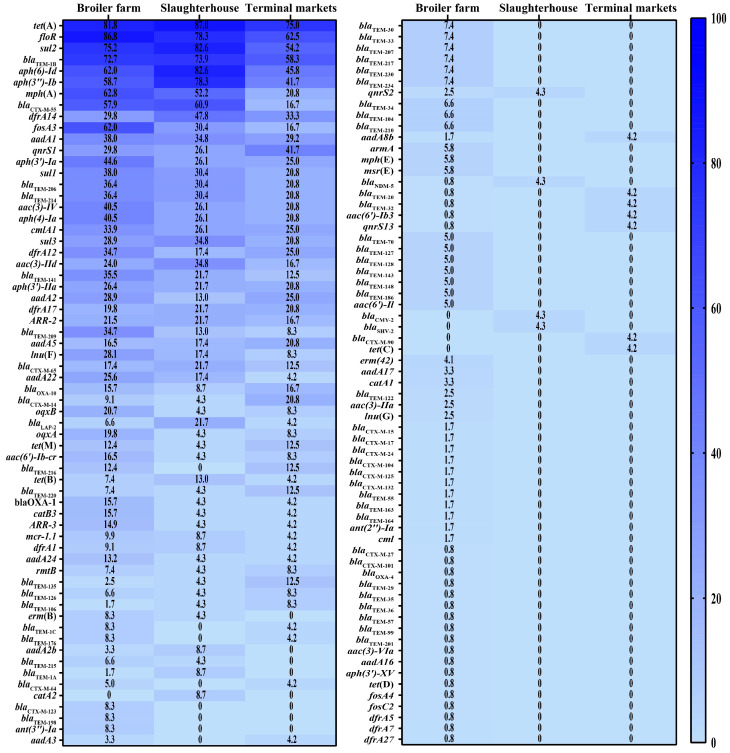
Heatmap of the antimicrobial resistance gene distributions in different *Escherichia coli* strains. The numbers in the cells correspond to the percentage of isolates that harbor plasmids.

In total, 129 catalogs of ARGs were detected among the 168 *E. coli* strains, and 1 *E. coli* isolate was not analyzed (Figure 2). The prevalences of *floR*, *tet*(A), *sul2*, *bla*_TEM-1B_, and *aph (6)-Id* were ≥60%. A total of 124 ARG catalogs were detected in *E. coli* strains from broiler farms. The prevalences of 89 ARG catalogs, such as *floR*, *mph*(A), and *fosA3*, were higher in the broiler farms than in the slaughterhouses and markets. Sixty ARG catalogs were detected in *E. coli* strains isolated from slaughterhouses. The prevalences of 21 ARG catalogs, such as *tet*(A), *sul2*, and *bla*_TEM-1B_, in the slaughterhouses were higher than those in the broiler farms and the markets. Sixty-three ARG catalogs were detected in *E. coli* strains from the markets. The prevalences of 19 ARG catalogs from the markets were higher than that in broiler farms and slaughterhouses, and these included *qnrS1*, *aadA5*, and *bla*_OXA-10_ (Figure 5). Carbapenem-resistance in two *E. coli* strains was mediated by *bla*_NDM-5_, and colistin-resistance in nine *E. coli* strains was mediated by *mcr-1* (Figure 2).

**Figure 6:**
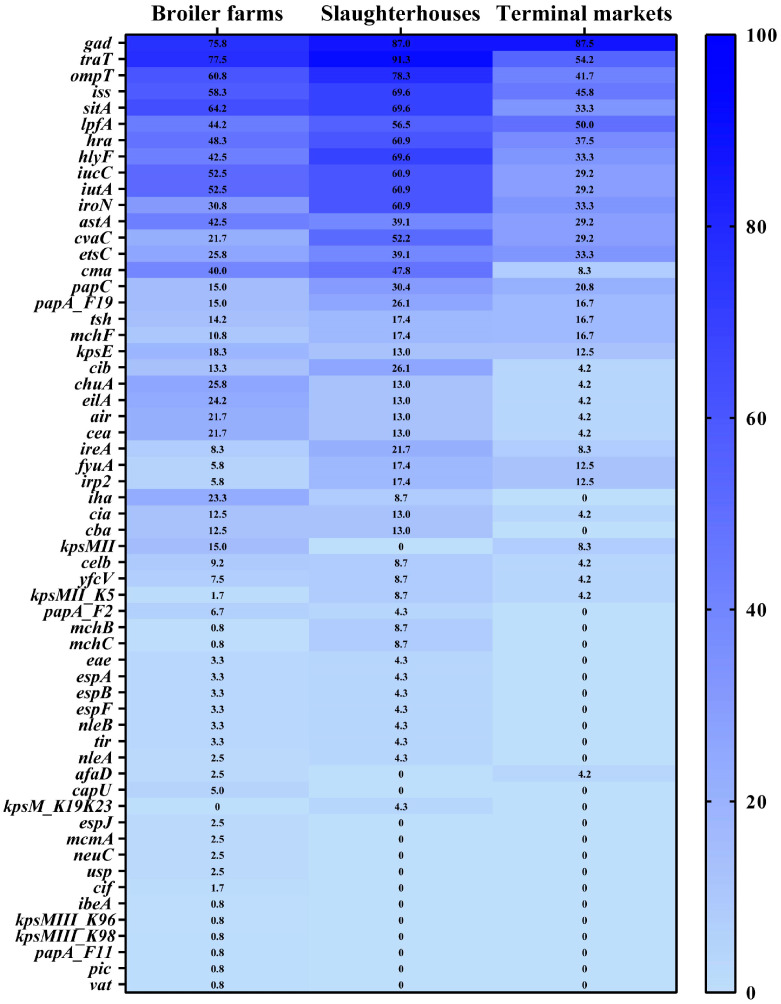
Heatmap of the virulence factor gene distributions in different *Escherichia coli* strains. The numbers in the cells correspond to the percentage of isolates that harbor plasmids.

In total, 60 catalogs of VFGs were detected among the 167 *E. coli* strains, and 2 *E. coli* isolates were not analyzed (Figure 6). The prevalences of *gad*, *traT*, *ompT*, and *sitA* were greater than 60%. A total of 58 VFG catalogs were detected in *E. coli* strains from the broiler farms. The prevalences of 22 catalogs of VFGs, such as *astA*, *kpsE*, and *chuA*, from the broiler farms were higher than those in the slaughterhouses and markets. Forty-five VFG catalogs were detected in *E. coli* strains isolated from the slaughterhouses. The prevalences of 35 VFGs, such as *traT*, *ompT*, and *iss*, from the slaughterhouses were higher than those in the broiler farms and markets. Thirty-four VFG catalogs were detected in *E. coli* strains from the markets (Figure 6). The prevalences of *gad* and *afaD* in the markets were higher than those in the broiler farms and slaughterhouses (Figure 6). Moreover, phylogenetic group A carried fewer VFGs than the other groups of *E. coli* (Figure 2).

### 3.5. Prevalence and Distribution of Plasmid Profiles

**Figure 7:**
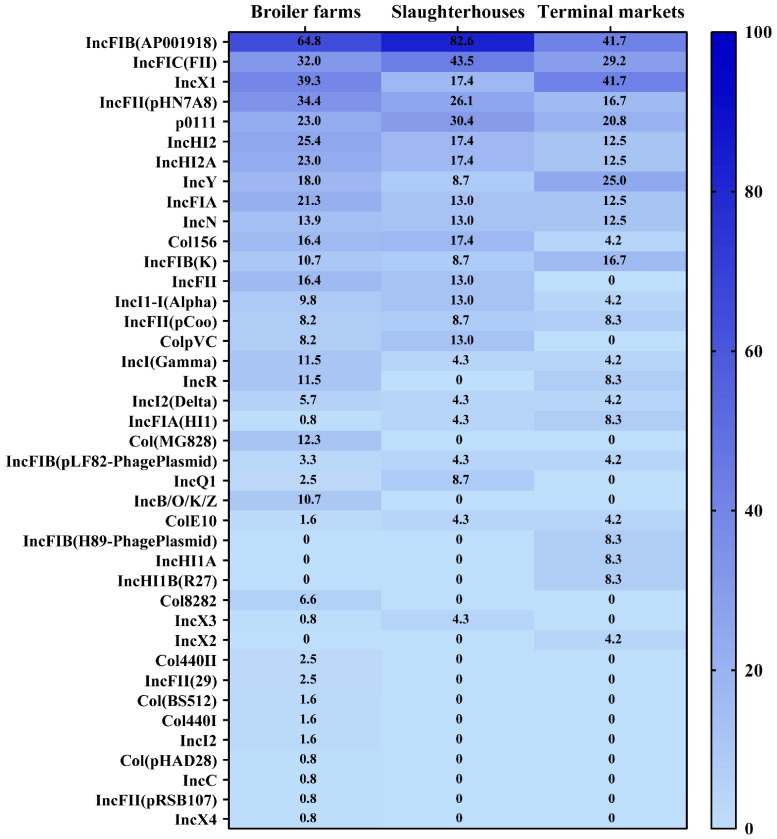
Heatmap of the plasmid distributions in different *Escherichia coli* strains. The numbers in the cells correspond to the percentages of isolates that harbor plasmids.

In total, 40 catalogs of plasmid replicons were detected among the 168 *E. coli* strains, and 1 *E. coli* isolate lacked plasmid (Figure 2). The IncFIB (AP001918), IncX1, IncFIC (FII), and IncFII (pHN7A8) plasmids were the most common, with prevalence rates of 63.9%, 36.7%, 33.1%, and 30.8%, respectively. Thirty-six plasmid catalogs were detected in *E. coli* strains from the broiler farms. The prevalences of 21 plasmid catalogs in the broiler farms were higher than that in the slaughterhouses and markets, and these included IncFII (pHN7A8), IncHI2, and IncHI2A. Twenty-three plasmid catalogs were detected in *E. coli* strains isolated from the slaughterhouses. The prevalences of 11 plasmid catalogs in the slaughterhouses were higher than those in the broiler farms and markets, and these included IncFIB (AP001918), IncFIC (FII), and p0111. Twenty-four plasmid catalogs were detected in *E. coli* strains isolated from terminal markets. The prevalences of eight plasmids, such as IncX1, IncY, and IncFIB (K), in the market were higher than those in the broiler farms and slaughterhouses (Figure 7).

## 4. Discussion

In this study, we compared the AMR phenotypes, STs, phylogenetic groups, O-serotypes, VFGs, ARGs, and plasmid replicons of *E. coli* isolated from the poultry industry in Henan province, China. The use of disinfectants in the environment, the manufacturing practices, and the nature of the samples might explain the differences in the prevalences of *E. coli*. MDR isolates are common in slaughterhouses [31,32]. In addition, contact between consumers and products of a similar nature to the samples could also have increased the prevalence. Wu et al. reported that retail chicken in China was commonly contaminated with *E. coli* that exhibit multiple drug resistance [33]. The finding that retail meat, specifically retail chicken meat, could be a reservoir for *E. coli* that cause human extraintestinal infections, as well as the substantial prevalence of human-pathogenic extraintestinal pathogenic *E. coli* (ExPEC)-associated genes and phenotypes among *E. coli* strains from retail chicken products, impelled us to focus more on food safety [34,35].

Our study of the AMR phenotypes of *E. coli* strains revealed that the presence of resistance genes was not always consistent with resistance to the corresponding antimicrobials. A similar result was demonstrated for Salmonella strains [36]. The presence of ARGs in chromosomes or their inability to be expressed could explain this. In addition, gene expression increased the MIC, but this was not sufficient to be defined as antibiotic-resistant bacteria. In 2016, the use of colistin to promote livestock growth was not permitted in China (http://www.moa.gov.cn/, accessed on 26 July 2016). It is possible that colistin-resistant strains have existed for a considerable period or may have emerged because of the noncompliant usage in violation of regulations. Compliance with these regulations by farms could potentially lead to a reduction in resistance rates. Peng et al. revealed the co-existence of *bla*_NDM-1_ and *mcr-1*, as well as other genes that are responsible for MDR phenotypes [37]. Lin et al. analyzed the genomic features of an *E. coli* ST156 strain isolated from a clinical patient harboring the chromosome-located *mcr-1* and plasmid-mediated *bla*_NDM-5_ gene [38]. An *E. coli* isolate named EC-YC1908-399 (ST10, O26) was found to carry both *bla*_NDM-5_ and *mcr-1* in this study, and further studies should be conducted to characterize the accurate structures of these plasmids.

ST131, ST69, and ST10 are the most common ST ExPEC strains [39]. The most widespread STs in poultry farms were determined to be ST48, ST155, and ST10 [40]. These results indicate that the STs are complicated and that *E. coli* ST10 is widespread. Similar results were observed in this study. ST10 is one of the most widespread STs in poultry supply chains. Importantly, ST10 could be associated with EPEC of various O-serogroups [41]. The discovery of an *E. coli* ST156 strain harboring a chromosome-located mcr-1 and plasmid-mediated blaNDM-5 gene strengthens the concern regarding ST156 [38]. Several STs can exist at two links in the poultry supply chain, and *E. coli* strains might spread throughout the broiler supply chain. The emergence of numerous completely different STs suggests that the poultry product was commonly contaminated by *E. coli* and that this contamination needs to be controlled.

*Escherichia coli* serogroups O169, O26, O145, O111, O103, O121, and O157 cause severe foodborne diseases [42,43,44,45]. Although numerous serotypes exist, they are not completely consistent. This suggests that *E. coli* strains have little potential to directly cause diseases. Although the prevalences of O21, O109, and O117 *E. coli* were too dispersed to reveal the possibility of *E. coli* infection along the broiler supply chain, this provides the targets for interventions to minimize bacterial spread. Moreover, bacteriophages and bacteriophage-derived endolysins have been proposed as alternative antibiotic strategies for improving food safety and public health [46,47].

These concerns are exacerbated by the high prevalence and abundance of acquired virulence genes, AMR genes, and plasmids. In general, there is a high risk of *E. coli* strains isolated from broiler farms and slaughterhouses disseminating plasmids harboring ARGs and virulence genes. The dominant acquired virulence genes were *gad*, *traT*, *ompT*, and *sitA*, of which *traT*, *ompT*, and *sitA* are related to ExPEC [35,48,49]. In this study, the dominant ARGs identified were *floR*, *tet*(A), *sul2*, *bla*_TEM-1B_, and *aph (6)-Id*. In the environment, *sul* and *tet* genes are abundant in manure, soil, and water samples [50]. These results could be related, and the transmission of these ARGs needs to be further analyzed. In one study, the prevalence of *tet*(A) and *bla*_TEM_ was ≥90% [51]. However, we did not observe a high prevalence in this study. The diversity of *E. coli* isolated from broiler farms, slaughterhouses, and sales terminals demonstrates that broiler farms and slaughterhouses have higher diversity and an increased risk. In this study, 40 different plasmid replicons were predicted, among which the Inc groups of the plasmids were the most common. Inc groups were reported to be the major plasmid families that emerged in MDR Enterobacteriaceae strains isolated worldwide, among those conferring resistance to clinically relevant antibiotics, and horizontal gene transfer through plasmids plays a major role in the evolution of AMR [52,53]. Removing plasmids from bacteria could be an important measure to fight AMR, and the utilization of plasmid-curing and anti-plasmid could reduce ARG prevalence, and sensitize bacteria to antibiotics [54].

Unfortunately, our study area did not involve too many cities and regions. In addition, our sample collection failed to track the changes in the process of chicken farm-to-table, which caused certain limitations. If there are more countries, provinces, or cities that monitored the prevalence of *E. coli*, data could be collected in a database, dynamic surveillance could be conducted, and the prevention and control of *E. coli* would be more targeted.

In summary, we characterized *E. coli* isolates from the broiler farms, slaughterhouses, and terminal markets of three companies in Henan province, China. Our results reveal that the different modes associated with terminal markets could cause different levels of bacterial contamination, and the environmental contamination of meat cannot be ignored. Therefore, the implementation of good manufacturing practices in the poultry industry is necessary to reduce the potential risk of *E. coli*.

## 5. Conclusions

The current study revealed that *E. coli* isolates from broiler farms, slaughterhouses, and terminal markets exhibited severe AMR profiles. Notably, some isolates were resistant to carbapenems, tigecycline, and colistin. Considering these severe AMR conditions, the use of antibiotics should be reduced particularly in broiler farms. Our results additionally supported that the STs and O-serotypes of these *E. coli* strains are extremely diverse and that the *E. coli* isolates from broiler farms and slaughterhouses harbor many ARGs and VFGs. The presence of those mobile plasmids will largely increase the AMR risk. In conclusion, our findings provide up-to-date information on the AMR profiles of *E. coli* from different sources in the poultry industry, and this information emphasizes the need to standardize antimicrobial use and good manufacturing practices, as well as sanitary conditions in related establishments.

## Data Availability

Data are contained within the article and Supplementary Materials.

## Supplementary Materials

The following supporting information can be downloaded at: https://www.mdpi.com/article/10.3390/microorganisms12030575/s1, Table S1: Primers used in this study; Table S2: Antimicrobial agents used in this study; Table S3: Minimal inhibitory concentrations of different antibiotics on *Escherichia coli*; Table S4: The isolation sources were the O-serogroups of 169 sequenced *Escherichia coli*.
